# Allopurinol therapy provides long term clinical improvement, but additional immunotherapy is required for sustained parasite clearance, in *L. infantum*-infected dogs

**DOI:** 10.1016/j.jvacx.2019.100048

**Published:** 2019-11-20

**Authors:** Leopoldo F.M. Nascimento, Dayane Francisca Higino Miranda, Luana D. Moura, Flaviane A. Pinho, Guilherme Loureiro Werneck, Ricardo Khouri, Steven G. Reed, Malcolm S. Duthie, Aldina Barral, Manoel Barral-Netto, Maria S.P. Cruz

**Affiliations:** a Universidade Federal do Piauí, Departamento de Morfofisiologia Veterinária, Teresina, PI, Brazil; b Universidade Federal da Bahia, Faculdade de Medicina, Salvador, BA, Brazil; c Universidade Federal do Rio de Janeiro, Instituto de Estudos em Saúde Coletiva, Rio de Janeiro, RJ, Brazil; d Fundação Oswaldo Cruz- Fiocruz, Instituto Gonçalo Moniz, Salvador, BA, Brazil; e Universidade Federal da Bahia, Faculdade de Medicina, Salvador, BA, Brazil; f Infectious Diseases Research Institute, Seattle, WA 98102, USA; g HDT Biotech Corporation, Seattle, WA 98102, USA; h Instituto Nacional de Ciência e Tecnologia, Instituto de Investigação em Imunologia, São Paulo, SP, Brazil

**Keywords:** Canine visceral leishmaniasis, Vaccine, Parasite, Drug, Clinical signs, CanL, canine leishmaniosis, GLA, glycopyranosyl lipid, IFN, interferon, IL, interleukin, MPL, monophosphoryl lipid A, SLA, Second generation Lipid Adjuvant, SE, stable emulsion, Th1, T helper 1-like cells, TLR, Toll-like receptor, VL, visceral leishmaniasis

## Abstract

There is little evidence that current control strategies for canine leishmaniosis (CanL), the veterinary disease caused by *L. infantum* infection, are having a positive impact. This is of critical importance because dogs are a primary reservoir for *L. infantum* and a significant source of parasite transmission to humans. Drugs intended primarily for human use are prohibited for the treatment of CanL because of concerns over the propagation of resistant parasites. Although allopurinol effectively decreases parasite burden in CanL the treatment needs to be maintained for life. We hypothesized that during the allopurinol-induced parasite reduction dogs may become capable of developing a more robust immune response that may permit more effective control of parasites. To test this, we investigated the clinical and parasitological impact of short-term treatment with allopurinol, either alone or in combination with a defined subunit vaccine, on dogs naturally infected with *L. infantum*. A total of 28 dogs were distributed as follows: untreated; oral allopurinol alone (20 mg/kg, once each day for 90 days); or allopurinol with immunization with the Leish-F2 antigen formulated with the Toll-like receptor (TLR) 4 agonist Second generation Lipid Adjuvant (SLA) in stable emulsion (SE; SLA-SE). Dogs that did not receive treatment had a progressive decline in their clinical condition and an increase in their infection levels, while treatment with allopurinol alone alleviated the clinical symptoms of CanL but did not generate sustained reduction in parasites. Concomitant immunization with Leish-F2 + SLA-SE, however, improved clinical condition while also providing long-term clearance of *L. infantum* from lymphoid tissues and systemic organs. These results have important implications for both the management of CanL and for limiting *L. infantum* transmission to humans.

## Highlights

• *L. infantum*-infected dogs were treated with allopurinol alone or plus Leish-F2 + SLA-SE.
• Both treatment regimen generated long term clinical improvement.
• Immunochemotherapy, but not chemotherapy alone, generated sustained parasite control.

## 1 Introduction

Visceral leishmaniasis (VL), the clinical consequence of infection with *Leishmnaia infantum*, is a serious public health problem in several countries. *L. infantum* can also infect dogs to cause canine leishmaniosis (CanL; also commonly called canine CVL), a multisystemic disease that manifests with a range of signs that can include dermatitis, lymphadenomegaly, general muscular atrophy and kidney failure. Dogs are incriminated as the main domestic reservoirs of *L. infantum* and are responsible for sustained transmission to humans [1]. Several measures have been recommended to combat CanL with a goal of limiting parasite transmission and disease in humans. In Brazil, due to the perceived risk of the potential emergence of drug resistant parasites, treatment of CanL is regulated by Interministerial Order No. 1426 that prohibits its treatment with drugs intended primarily for human use [2]. Given that currently available chemotherapies have only been partially effective, several alternative treatment options have been investigated [2]. For example, vaccines can be used more broadly than drugs, and could be used to protect both animals and humans. The vaccines used for *Leishmania*, have, however, demonstrated varying efficacies when evaluated by prophylactic or therapeutic approaches [3].

When appropriately adjuvanted the recombinant fusion protein Leish-111f (L111f; LEISH-F1; MML) promotes protective immunity in mice, hamsters and rhesus monkeys [4], [5]. LEISH-F1 + monophosphoryl lipid A (MPL)-SE was the first subunit vaccine against *Leishmania* to enter human clinical trials [6]. Subsequent modifications of the Leish-F1 polyprotein were made to simplify manufacturing, with the modified construct renamed Leish-110f (Leish-F2) [7].

Concurrent with advances in molecular biology techniques that have made protein production more efficient, the development of defined adjuvants has provided a major additional breakthrough in vaccine development. Adjuvants can now be designed to enhance and bias T cell responses, and Toll-like receptor (TLR) agonists have been extensively used to promote antigen-specific T helper (Th) 1 immune responses that are required for protection against intracellular pathogens such as *Leishmania* [8]. In particular, monophosphoryl lipid A (MPL), a multiple species TLR4 agonist extracted from *Salmonella*, has a long history of incorporation in approved vaccines [9]. Single species synthetic TLR4 agonists such as glycopyranosyl lipid A (GLA) and second generation lipid adjuvant (SLA) have now been developed and advanced into human clinical trials [10].

This study was conducted to determine if immunization could be used in conjunction with drug therapy to improve the clinical and parasitological outcome of CanL. For the chemotherapeutic component we used allopurinol, a drug previously used in the treatment of dogs infected with *L. infantum*. For the vaccine component we used the Leish-F2 polyprotein and contrasted its impact when formulated with an adjuvant that has the ability to stimulate TLR4 (the second-generation lipid adjuvant in stable emulsion (SLA-SE)).

## 2 Materials and methods

### 2.1 Study population

The study was conducted in the Experimentation Kennel and in the Laboratory of Infectious Diseases, Center of Agricultural Sciences, University Veterinary Hospital of the Universidad Federal do Piaui (UFPI). Twenty eight dogs, all naturally infected with *L. infantum*, were obtained from the Management of Zoonoses of the Municipal Health Foundation of Teresina, Piaui or from University Veterinary Hospital, UFPI. Dogs were obtained by donation from their original owner after the study objectives were described to them and after they had signed the donation agreement. Collectively the dogs were adults of either gender, unknown age, and of various defined or undefined breeds. Animals were excluded if they were diagnosed with *Ehrlichia canis* (Alere Erliquiose Ac test KIT®) and canine distemper (Alere Cinomose Ag test kit®, Sao Paulo, Brazil). Animals were included based on the positive identification of *L. infantum* in aspirates of lymph node, skin and/or bone marrow, and if they were amenable to oral treatment and collection of samples. Each animal was provided an identification tag by subcutaneous implantation of a microchip, vermifugation (Dauverm Plus, Vansil®) and immunization against common viruses (Vanguard HTLP 5/CV-L; Pfizer Animal Health, New York, USA) before housing in screened kennels measuring 1.5 m width/3.0 m length. Water and food were provided *ad libitum*. All procedures were conducted in accordance with the recommendations of the Brazilian College of Animal Experimentation (COBEA).

### 2.2 Experimental groups

**Fig. 1:**
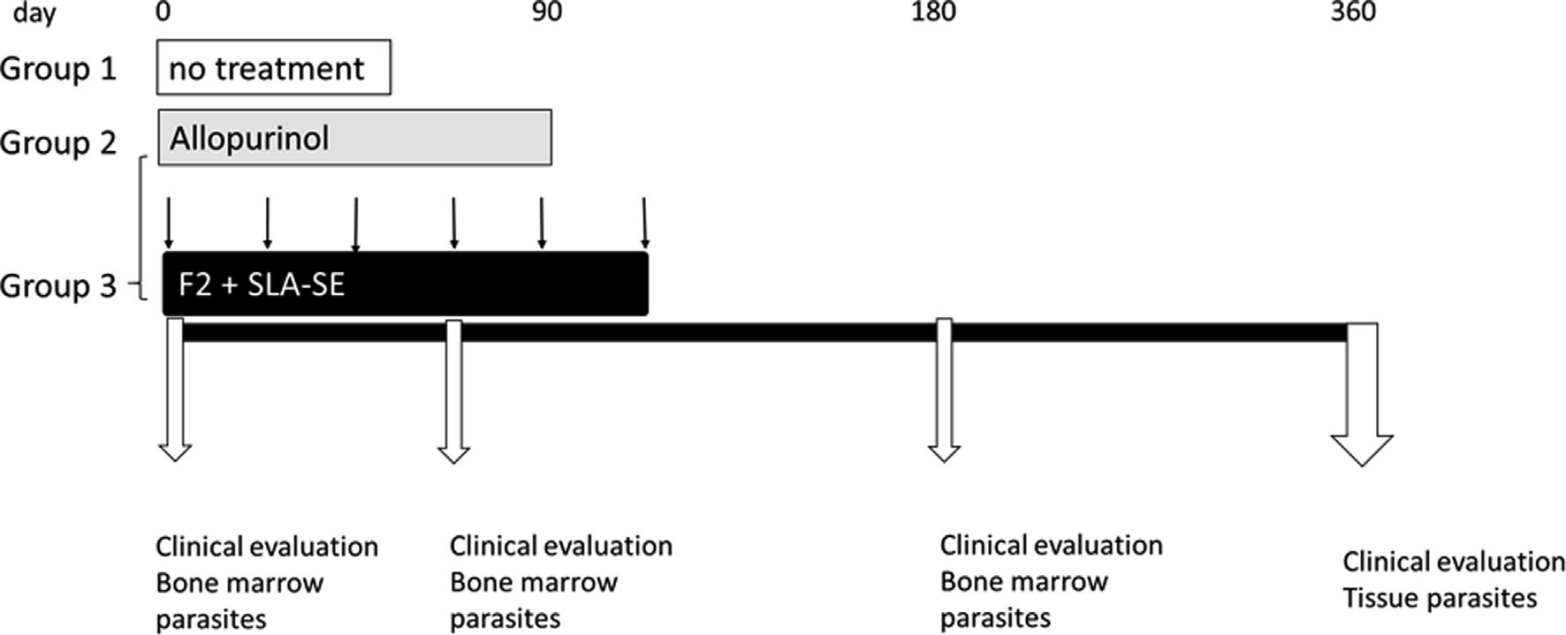
Schematic of study design. Dogs were confirmed to be infected with *L. infantum* and provided a full clinical examination to generate a clinical score for each animal before placement into treatment groups. A group of 6 dogs did not receive medication (untreated), while all other animals received allopurinol therapy orally at a dose of 20 mg/kg, once each day, for 90 days. A group of 8 dogs received allopurinol therapy alone (allopurinol); 8 dogs were additionally subcutaneously injected with the Leish-F2 + SLA-SE for a total of 6 doses, with a 3 week interval between each dose (Allo plus Leish-F2 + SLA-SE). Dogs were monitored throughout and full evaluations conducted to yield clinical scores.

Dogs were allocated to treatment groups by a stratified randomization procedure that was conducted to ensure comparability between the groups. Four strata were created based on the median of the two variables: parasite load in bone marrow aspirate and clinical score. Each group contained animals with: (1) high parasite load and high clinical score; (2) high parasite load and low clinical score; (3) low parasite load and high clinical score; or (4) low parasite load and low clinical score. Within each stratum the animals were then randomly allocated to a treatment group. Group 1 comprised 6 dogs that did not receive medication, while Groups 2 and 3 each received oral allopurinol at a dose of 20 mg/kg, one time each day for 90 days. Group 2 comprised 8 dogs that received only allopurinol; Group 3 comprised 8 dogs that were also subcutaneously injected with Leish-F2 + SLA-SE (20 μg each) for a total of 6 doses, with a 3 week interval between each dose (Leish-F2 lot WR823-1370, SLA-SE lot #QG307; both IDRI). A schematic of the experimental design is shown in Fig. 1.

### 2.3 Clinical evaluation of animals

Each dog was clinically evaluated and scores assigned as previously described [11]. A variety of signs attributable to *Leishmania* infection (and relative score) were evaluated in each dog. Systemic signs evaluated and assigned scores were: Attitude: active (0), apathetic (1); Ectoparasites: absence (0), fleas (1), fleas and ticks (2); Nutritional status: normal (0), thin (1), cachectic (2); Lymph nodes: normal (0), enlarged (1); Mucosal coloring: normal (0), pale (1); Bleeding: absence (0), presence (1). Cutaneous signs evaluated and assigned scores were: Bristles: good (0), regular (1), bad/opaque (2); Muzzle/ear lesion: absence (0), presence (1); Nails: normal (0), long/onychogryphosis (1); Skin lesion: absence (0), presence (1), ulcer (2); Muzzle depigmentation: absence (0), presence (1); Alopecia: absence (0), presence (1). Ocular signs evaluated and assigned scores were: Blepharitis: absence (0), presence (1); Keratoconjunctivitis: absence (0), serous (1), mucopurulent (2). The clinical assessment was performed by three veterinarians who scored the signs according to the above criteria. Individual scores were added to produce a total sign-based score ranging between 0 and 19, adapted from Manna *et al*. [12].

### 2.4 Sample collection

Bone marrow aspirates were collected on days 0, 63, 180 and 360 by physically restraining each animal and inserting a needle into the sternum to slowly extract 1.5 mL of marrow. At the end of the monitoring period all dogs underwent sedation by intravenous injection of xyzaline hydrochloride (1 mg/kg) and ketamine hydrochloride (10 mg/kg), and after 10 min, the general anesthetic 2.5% sodium thiopental was administered intravenously at the dose of 0.5 mL/kg. After reaching the appropriate anesthetic plane, T-61 (Hoechst Roussel Vet) was administered and death was confirmed by the absence of heart beat and respiration. Samples were collected from the spleen, liver, kidney, skin and lymph nodes.

### 2.5 Determination of infection levels as parasite genome equivalents

DNA was extracted using the PureLink® Genomic DNA Minikit (Invitrogen™ Cat No. 1820-02), following the manufacturer's recommendations for each particular sample type. DNA quantity was determined using Nanodrop™ 2000/2000c spectrophotometer (Thermo Scientific), with purity evaluated by the 260 nm/280 nm ratio. The quality of the extracted DNA was evaluated by conventional PCR amplifying the housekeeping gene beta-actin with the following primers: Primer β-Actin Forward GACCCTGAAGTACCCCATTGAG and Primer β-Actin Reverse TTGTAGAAGGTGTGGTGCCAGAT (Invitrogen®, São Paulo, Brazil). For each PCR reaction, 20 ng DNA was amplified using 2 mM MgCl_2_, 200 μM dNTPs, 0.6 μM of each primer, 1 IU Platinum Taq DNA Polymerase and specific buffer (all Invitrogen). The thermocycling program was 95 °C for 5 min followed by 35 cycles of 30 s by 95 °C; 58.8 °C for 45 s, 72 °C for 45 s; and final extension at 72 °C for 7 min. The amplification product was then observed on 1.5% agarose gel stained with ethidium bromide.

Infection levels were determined as parasite genome equivalents by real-time PCR according to a previously described protocol (MANNA et al., 2008). Each sample was evaluated in duplicate. *L. infantum* DNA was amplified using the primers: sense-5′-GGTTAGCCGATGGTGGTCTT-3 'and anti-sense – 5′-GCTATATCATATGTCCAAGCACTTACCT-3′ and TaqMan probe (5′-ACCACCTAAGGTCAACCC- . In brief, 5 μL of DNA sample (100 ng) was added to 16 μL of a mixture consisting of 12.5 μL of 2x TaqMan Universal PCR Master Mix, No AmpErase UNG (Applied Biosystems), 1.25 μL of each primer, 1.0 μL TaqMan MGB probe (FAMTM dye-labeled, Applied Biosystems) and 4 μL of milli-Q water, to provide a 25 μL volume for each reaction. Amplification was performed on an ABI 7500 real time PCR system using SDS 2.0 software (Applied Biosystems) with denaturing at 95 °C for 10 min, followed by 40 cycles of 95 °C for 15 s and 60 °C for 1 min. Quantification was achieved by extrapolation into a standard curve generated from a range of 1 to 1 × 10^6^ cultured *L. infantum* parasites (MCAN/BR/89/Ba-262) [13].

### 2.6 Statistical analysis

D'Agostino & Pearson normality test, Shapiro-wilk normality test and KS normality test determined the choice of non-parametric graphs types and testing. Superimposed symbols at median with interquartile range with connecting line, Box and Whisker plot or Heatmap were used to represent data distribution. A two-tailed Kruskal–Wallis test with Dunn's multiple comparison post-test were used to compare three or more unmatched groups. All statistical tests were two-tailed with a significance level of 5% (GraphPad Prism 7.0 software).

### 2.7 Data availability

The underlying data reported in this paper are available from the corresponding author upon reasonable request.

## 3 Results

### 3.1 Treatment with either drug or drug plus vaccine prevents clinical progression of CanL

**Fig. 2:**
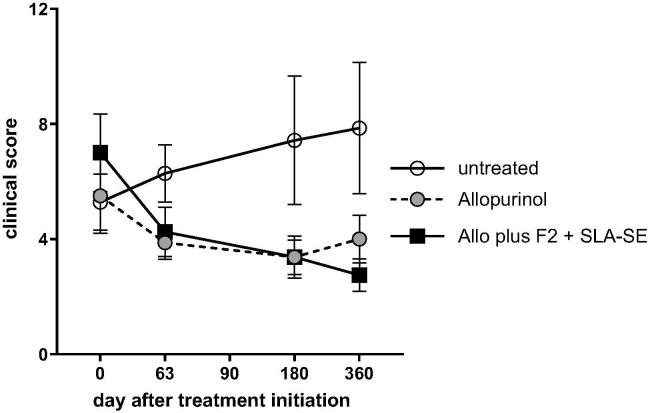
Allopurinol, either alone or in combination with F2 + SLA-SE, alleviates clinical signs of canine visceral leishmaniasis (CVL). Dogs were confirmed to be infected with *L. infantum* and provided a full clinical examination to generate a clinical score for each animal before placement into treatment groups. A group of 6 dogs did not receive medication (untreated), while all other animals received allopurinol therapy orally at a dose of 20 mg/kg, once each day, for 90 days. A group of 8 dogs received allopurinol therapy alone (allopurinol); 8 dogs were additionally subcutaneously injected with the Leish-F2 + SLA-SE (Allo plus Leish-F2 + SLA-SE). Vaccine was provided for a total of 6 doses, with a 3 week interval between each dose. Dogs were monitored throughout and full evaluations conducted at the indicated times to yield clinical scores. Results are shown as line plot, with the symbol representing the median and vertical error bars representing the interquartile range (Group comparison of clinical score: Kruskal-Wallis test, *p*-value < 0.05 and Dunn's comparative post-test, *p*-value < 0.0332).

To evaluate if chemo- and or chemoimmunotherapeutic intervention could interrupt *L. infantum* infection and CanL development, we enrolled dogs that were confirmed to be infected with *L. infantum*. No side effects were observed with either allopurinol alone or allopurinol plus Leish-F2-SLA-SE treatment. As expected, animals that did not receive treatment exhibited a steady worsening in their clinical score, indicating the natural progression of the disease (Fig. 2). In contrast, animals receiving allopurinol alone or in combination with Leish-F2 + SLA-SE demonstrated an improvement in clinical response that extended even after completion of treatment, with all treated dogs maintaining a low clinical score throughout the monitoring period (Fig. 2). Together, these data indicate successful intervention in the clinical advancement of CanL.

### 3.2 Treatments reduce infection within bone marrow but F2 + SLA-SE immunization is required for sustained parasite control

**Fig. 3:**
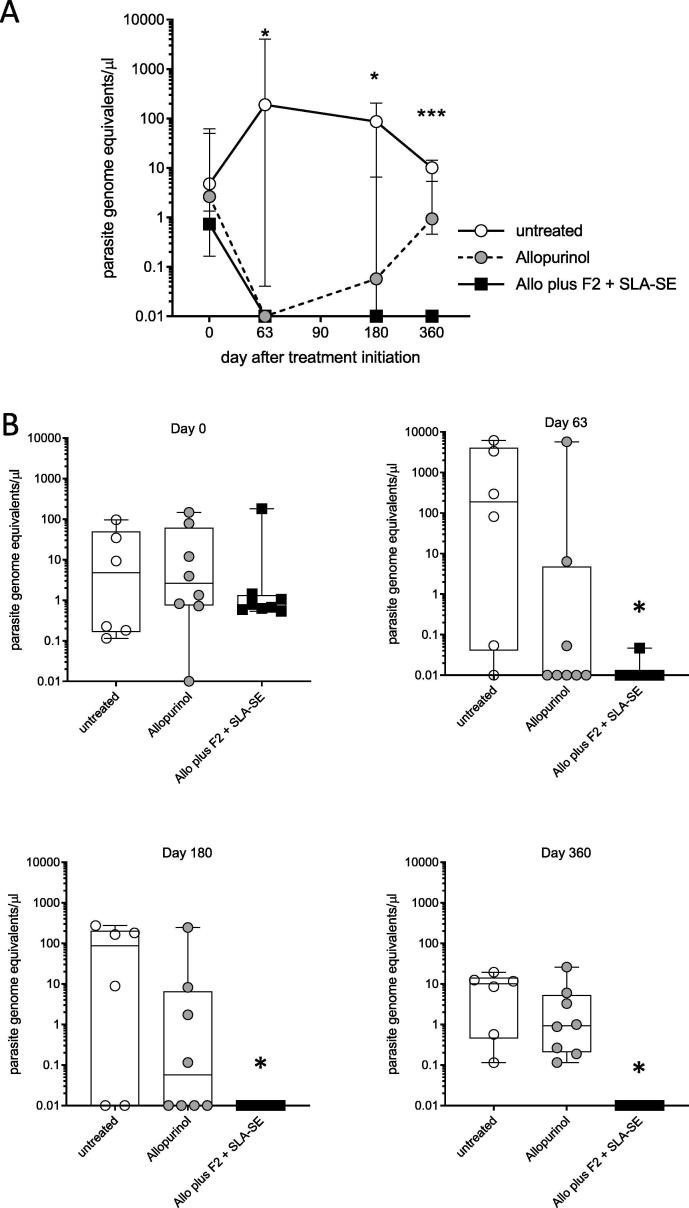
Combined treatment with allopurinol and F2 + SLA-SE is required for sustained parasite clearance from the bone marrow of *L. infantum*-infected dogs. Bone marrow aspirates were collected from dogs before, and at intervals after, therapeutic intervention. *L. infantum* genome equivalents were determined by real time PCR. Animals were either not treated (n = 6) or were treated with allopurinol alone (n = 8) or allopurinol plus Leish-F2 + SLA-SE (n = 8). In (A), dogs were monitored throughout and real time PCR was conducted to quantify parasite genomes equivalents. Results are shown as line plot with the symbol marking the median and vertical error bars representing the interquartile range. In (B), results from each individual dog are displayed for each analyses time (indicated above each plot). Each point shows the parasite number detected for an individual animal, with the Box and Whisker representing the median, the lower and upper quartiles, and the maximum and minimum values per group. * = *p*-value < 0.05 versus untreated group.

Having demonstrated that treatment improved the clinical condition of *L. infantum*-infected dogs, we then evaluated if this was associated with reduced infection levels (assessed as parasite genome equivalents). Given that *L. infantum* replicates in the lymphoid organs, we assessed infection within bone marrow. At the beginning of the treatment, each group had low, but similar, parasite numbers (Fig. 3A and B). Consistent with their symptomatic progression, dogs that received no treatment generally exhibited expansion of parasites within the first 2 months and retained detectable parasites throughout the 1-year monitoring period (Fig. 3). Close evaluation revealed that oral allopurinol monotherapy cleared parasites to below detectable levels in only 5 of the 8 dogs by day 63 after treatment initiation (Fig. 3C) and all eight dogs had detectable parasites one year after treatment (Fig. 3E). At each time assessed after treatment initiation there was a significantly lower number of *L. infantum* in the bone marrow of dogs that received allopurinol plus Leish-F2 + SLA-SE relative to those quantified in untreated dogs (*p*-values = 0.0145, 0.0267 and 0.0017 at days 63, 90 and 360, respectively) (Fig. 3). By day 360 statistically significant differences were observed between dogs provided allopurinol with Leish-F2 + SLA-SE and dogs that received allopurinol alone (Fig. 3E; *p*-value = 0.0058). In contrast, addition of Leish-F2 + SLA-SE to the allopurinol regimen provided a long term, 1 year clearance of parasites to levels below the limit of detection in all but one of the immunized animals (Fig. 3E). Taken together, these data indicate that while treatment with allopurinol alone provided a transient reduction of parasite burdens, addition of Leish-F2 + SLA-SE was required for the sustained elimination of *L. infantum* from the bone marrow.

### 3.3 Immunization provides sustained protection against parasite dissemination

**Fig. 4:**
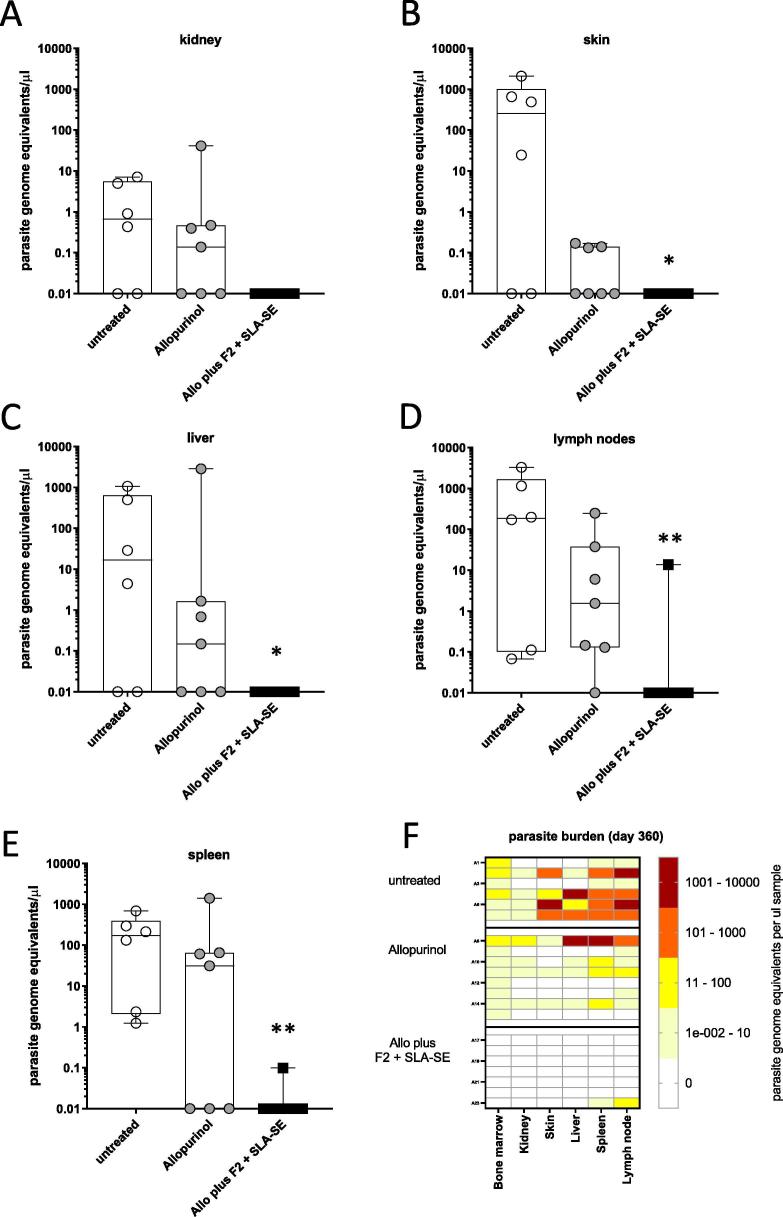
Combined treatment with allopurinol and F2 + SLA-SE is required for sustained parasite clearance from lymphoid and non-lymphoid organs of *L. infantum*-infected dogs. Dogs were either not treated (n = 6) or were treated with allopurinol alone (n = 8) or allopurinol plus Leish-F2 + SLA-SE (n = 8). Samples were collected at termination of the observation period and *L. infantum* burden determined by real time PCR. Parasite burdens were determined in samples from (A) kidney, (B) skin, (C) liver, (D) lymph nodes and (E) spleen. Each point shows the parasite number detected for an individual animal, with the Box and Whisker overlay representing the median, the lower and upper quartiles, and the maximum and minimum values per group. * and ** = *p*-values < 0.05 and 0.01 versus untreated group, respectively. In (F), a heatmap showing parasite genome equivalents for each animal is shown.

To determine if parasite dissemination had been impacted, other tissues were collected at the termination of the monitoring period. Parasites were found in the lymph node and spleen samples of all of the untreated animals, while a proportion of the untreated dogs also displayed infection in their liver, kidney, and skin samples (Fig. 3). Parasites were also detected in the other organs collected from dogs treated with allopurinol alone. In contrast, parasites were undetectable in organs from all but one of the dogs treated with allopurinol plus Leish-F2 + SLA-SE (a low number of parasites were detected in the spleen and lymph node samples from this single animal) (Fig. 4). Taken together, these data indicate that treatment with allopurinol plus Leish-F2 + SLA-SE provides a robust, sustained systemic reduction of *L. infantum* from infected dogs.

## 4 Discussion

Monotherapy with allopurinol is one of the preferred treatments for CanL. In this study, we observed that allopurinol, either alone or in conjunction with defined subunit vaccine Leish-F2 + SLA-SE, provided long-term clinical improvement of the infected dogs. When we evaluated the infectious status, however, our data revealed that although treatment with allopurinol alone initially reduced parasite burden in the bone marrow, one year later numbers had rebounded to pre-treatment levels and infection was disseminated throughout the body. In contrast, addition of Leish-F2 + SLA-SE to the allopurinol treatment generated a prolonged clearance of parasites from lymphoid and non-lymphoid organs. These results demonstrate an improved treatment regimen for CanL and strongly suggest the potential for inhibiting transmission of *L. infantum* from the canine reservoir in endemic regions. As such, these results have major implications for both CanL and human VL control programs.

In agreement with earlier reports that long-term allopurinol monotherapy generates a good clinical response, dogs treated with allopurinol alone responded well and demonstrated clinical improvement. We did not observe the clinical relapse that has been reported at a rate of up to 88.9% within 2–4 weeks of allopurinol withdrawal [14]. Consistent with results observed with other drugs such as miltefosine and paramomycin, is apparent from our results that short-term allopurinol chemotherapy alone cannot control the infection in the long term and that parasites can reemerge after treatment is removed [15]. Persistence of parasites long after clinical ‘cure’ of CanL by chemotherapy leaves many treated dogs still infective to sand flies for months after treatment completion [16]. This persistence is apparent in our study because, despite clinical responsiveness and alleviation of symptoms, one year after treatment initiation we observed parasites in the bone marrow of each animal treated with only allopurinol. At study termination parasite dissemination to lymphoid organs (bone marrow, lymph node, spleen) was observed, probably due to infection of phagocytic cells (macrophages and dendritic cells) for which *Leishmania* exhibits tropism [17]. Parasites were also detected in kidneys, liver and skin, indicating widespread systemic dissemination. Although we did not formally examine transmission potential by xenodiagnostic procedures, the presence of parasites in the skin likely means that they are available for uptake during the blood meal of sand flies [18], [19]. In contrast to chemotherapy alone, the return of parasites was observed at only low levels in only one of the dogs that had been immunized F2 + SLA-SE in addition to their allopurinol treatment. Taken together these data suggest that while allopurinol generates an immediate, early restraint on the advancement of symptoms and parasite replication, the addition of immunization provides a response that likely promotes their clearance and a decreased transmission potential.

Immunization has been previously studied as an immunotherapy for CanL, with differing outcomes. Therapeutic administration of total antigens of Leishmania amazonensis plus saponin (LaSap) to naturally infected dogs promoted clinical cure and did reduce parasite loads in the skin for at least 180 days [20]. Investigation of a vaccine composed of antigens of *Leishmania braziliensis* associated with MPL adjuvant (LBMPL vaccine) for CanL revealed that dogs provided therapy with LBMPL demonstrated reductions in the number and intensity of the disease signs as well as parasite burdens assessed by real-time PCR. LBMPL immunotherapy promoted a blockade in transmission of *L. infantum* to sand flies, with only 3 of 9 dogs being positive by xenodiagnosis 90 days after treatment [21]. Although neither compatibility with drug regimen nor parasite levels were investigated, it was recently reported that providing LeishTec® (recombinant Leishmania A2 protein + saponin) in an immunotherapeutic regimen reduced the risk of progression to CanL by 25% in all asymptomatic dogs regardless of age and reduced mortality rates in asymptomatic dogs 6 years of age or younger by 70% [22]. Supplementation with the TLR4 agonist MPL to subunit vaccines has promoted success, or contrarily has had no impact, in halting disease progression [23], [24], [25]. The differing outcomes of these studies could, however, have arisen because infection levels were not quantified before the start of treatment. It is well established that infection and clinical status of each particular dog influences the response to treatment, particularly as that the onset of immune exhaustion precedes the transition from asymptomatic *Leishmania* infection to progressively worsening CanL [26]. This immune exhaustion is characterized by anti-inflammatory responses, lack of proliferation in response to *Leishmania* antigens, and lymphocyte apoptosis [27]. *Ex vivo* incubation with combinations of TLR agonists indicate the potential to overcome *L. infantum*-induced cellular exhaustion, allowing robust Th1 responses in cells from symptomatic dogs that exhibit dampened responses to antigen alone [28]. The initial use of chemotherapy to transiently reduce parasite burden may permit immune responsiveness that can be manipulated by immunization to generate sustained parasite control. It remains to be determined how exactly our chemo-immunotherapeutic achieves long term clinical and parasitological improvements.

Leish-F2 has previously been evaluated in combination with MPL-SE vaccine and Glucantime chemotherapy to treat canine visceral leishmaniasis. Parasite presence was evaluated by smear of the ear skin biopsies, bone marrow smears, bone marrow cultures, and entomological PCR from infected sand flies before treatment then again at days 90 and 180 after therapy, finding parasites in samples from 3 of the 5 surviving dogs that received chemoimmunotherapy [24]. The success of the Leish-F2 + SLA-SE vaccine used in our study could potentially be attributed to the use of the refined TLR4 agonist, that was generated through modifications of acyl chains of GLA, which in itself is a second-generation synthetic single species evolution of *S. minnesota*-derived monophosphoryl lipid A (MPL) [10]. SLA is capable of inducing Th1 profile cytokines while maintaining low levels of anti-inflammatory cytokines. It is well documented that TLR4 agonists can act synergistically when used simultaneously with other TLR agonists such as imiquimod, leading to elevated levels of IFNγ and IL-12 that would enhance anti-parasitic responses [29]. Only mice treated with Leish-F2 plus a combination of monophosphoryl lipid A-CpG, but nto either TLR agonsit alone, were able to induce a strong effective antigen-specific T cell response during therapy of *L. major* infection of mice; drug treatment was not included in that study [30]. In our study the use of a combination SLA/imiquimod-SE adjuvant did not appear to provide as much benefit as the use of SLA-SE alone (data not shown). The group of animals that received allopurinol plus Leish-F2 + SLA-SE chemoimmunotherapy demonstrated statistically significant reductions in parasite burdens in bone marrow at all times following treatment initiation, with the sustained reduction at day 360 being significantly lower than the burden of animals treated with allopurinol only chemotherapy. It is evident that the inclusion of Leish-F2 + SLA-SE immunizations facilitated the long-term clearance of parasites from most dogs. Variables such as vaccine dose and administration frequency, along with the adjuvant formulation used, could all influence the outcome and further analyses of these parameters appears merited before broader application.

In conclusion, the present study presents promising results indicating that use of a combined chemoimmunotherapeutic approach, involving allopurinol treatment in conjunction with the Leish-F2 + SLA-SE vaccine formulation, for the treatment of CanL. This chemoimmunotherapy of alleviated symptoms and reduced *L. infantum* burdens in naturally infected dogs in both the short- and long-term. As such, these results represent a major advance in the treatment and management of CanL, as well as the management of *L. infantum* infection and potential transmission to cause human disease.

## Author contributions

Experimental design: Leopoldo Fabrício Marçal do Nascimento, Dayane Francisca Higino Miranda, Luana Dias de Moura, Flaviane Alves de Pinho, Guilherme Loureiro Werneck, Ricardo Khouri, Steven G. Reed, Malcolm S. Duthie, Aldina Maria do Prado Barral, Manoel Barral-Netto, Maria do Socorro Pires e Cruz

Veterinary procedures: Leopoldo Fabrício Marçal do Nascimento, Dayane Francisca Higino Miranda, Luana Dias de Moura, Flaviane Alves de Pinho, Maria do Socorro Pires e Cruz

Laboratory procedures: Leopoldo Fabrício Marçal do Nascimento, Dayane Francisca Higino Miranda, Luana Dias de Moura, Flaviane Alves de Pinho, Ricardo Khouri, Maria do Socorro Pires e Cruz

Data analyses: Leopoldo Fabrício Marçal do Nascimento, Luana Dias de Moura, Guilherme Loureiro Werneck, Ricardo Khouri, Aldina Maria do Prado Barral, Manoel Barral-Netto, Maria do Socorro Pires e Cruz

Manuscript preparation: Leopoldo Fabrício Marçal do Nascimento, Luana Dias de Moura, Guilherme Loureiro Werneck, Ricardo Khouri, Steven G. Reed, Malcolm S. Duthie, Aldina Maria do Prado Barral, Manoel Barral-Netto, Maria do Socorro Pires e Cruz

## Declaration of Competing Interest

The authors declare the following financial interests/personal relationships which may be considered as potential competing interests: [Malcolm Duthie and Steven Reed are co-inventors on a patent for leishmaniasis vaccine development. The other authors have stated no conflict of interest.].

## References

[1] Travi B.L. (2014). Ethical and epidemiological dilemmas in the treatment of dogs for visceral leishmaniasis in Latin America. Biomedica.

[2] Noli C., Auxilia S.T. (2005). Treatment of canine Old World visceral leishmaniasis: a systematic review. Vet Dermatol.

[3] Ghorbani M., Farhoudi R. (2018). Leishmaniasis in humans: drug or vaccine therapy?. Drug Des Devel Ther.

[4] Campos-Neto A., Porrozzi R., Greeson K., Coler R.N., Webb J.R., Seiky Y.A. (2001). Protection against cutaneous leishmaniasis induced by recombinant antigens in murine and nonhuman primate models of the human disease. Infect Immun.

[5] Skeiky Y.A., Coler R.N., Brannon M., Stromberg E., Greeson K., Crane R.T. (2002). Protective efficacy of a tandemly linked, multi-subunit recombinant leishmanial vaccine (Leish-111f) formulated in MPL adjuvant. Vaccine.

[6] Chakravarty J., Kumar S., Trivedi S., Rai V.K., Singh A., Ashman J.A. (2011). A clinical trial to evaluate the safety and immunogenicity of the LEISH-F1+MPL-SE vaccine for use in the prevention of visceral leishmaniasis. Vaccine.

[7] Bertholet S., Goto Y., Carter L., Bhatia A., Howard R.F., Carter D. (2009). Optimized subunit vaccine protects against experimental leishmaniasis. Vaccine.

[8] Reed S.G., Bertholet S., Coler R.N., Friede M. (2009). New horizons in adjuvants for vaccine development. Trends Immunol.

[9] Garcon N., Van Mechelen M. (2011). Recent clinical experience with vaccines using MPL- and QS-21-containing adjuvant systems. Expert Rev Vaccines.

[10] Carter D., Fox C.B., Day T.A., Guderian J.A., Liang H., Rolf T. (2016). A structure-function approach to optimizing TLR4 ligands for human vaccines. Clin Transl Immunol.

[11] Silva K.R., Mendonca V.R., Silva K.M., Nascimento L.F., Mendes-Sousa A.F., Pinho F.A. (2017). Scoring clinical signs can help diagnose canine visceral leishmaniasis in a highly endemic area in Brazil. Mem Inst Oswaldo Cruz.

[12] Manna L., Reale S., Vitale F., Gravino A.E. (2009). Evidence for a relationship between Leishmania load and clinical manifestations. Res Vet Sci.

[13] de Paiva Cavalcanti M., Felinto de Brito M.E., de Souza W.V., de Miranda Gomes Y., Abath F.G. (2009). The development of a real-time PCR assay for the quantification of Leishmania infantum DNA in canine blood. Vet J.

[14] Cavaliero T., Arnold P., Mathis A., Glaus T., Hofmann-Lehmann R., Deplazes P. (1999). Clinical, serologic, and parasitologic follow-up after long-term allopurinol therapy of dogs naturally infected with Leishmania infantum. J Vet Intern Med.

[15] Miro G., Petersen C., Cardoso L., Bourdeau P., Baneth G., Solano-Gallego L. (2017). Novel areas for prevention and control of canine leishmaniosis. Trends in Parasitology.

[16] Alvar J., Molina R., San Andres M., Tesouro M., Nieto J., Vitutia M. (1994). Canine leishmaniasis: clinical, parasitological and entomological follow-up after chemotherapy. Ann Trop Med Parasitol.

[17] Reis A.B., Martins-Filho O.A., Teixeira-Carvalho A., Carvalho M.G., Mayrink W., Franca-Silva J.C. (2006). Parasite density and impaired biochemical/hematological status are associated with severe clinical aspects of canine visceral leishmaniasis. Res Vet Sci.

[18] Pereira-Fonseca D.C.M., Oliveira-Rovai F.M., Rodas L.A.C., Beloti C.A.C., Torrecilha R.B.P., Ito P. (2017). Dog skin parasite load, TLR-2, IL-10 and TNF-alpha expression and infectiousness. Parasite Immunol.

[19] Courtenay O., Carson C., Calvo-Bado L., Garcez L.M., Quinnell R.J. (2014). Heterogeneities in Leishmania infantum infection: using skin parasite burdens to identify highly infectious dogs. PLoS Negl Trop Dis.

[20] Viana K.F., Lacerda G., Teixeira N.S., Rodrigues Cangussu A.S., Sousa Aguiar R.W., Giunchetti R.C. (2018). Therapeutic vaccine of killed Leishmania amazonensis plus saponin reduced parasite burden in dogs naturally infected with Leishmania infantum. Vet Parasitol.

[21] Roatt B.M., Aguiar-Soares R.D., Reis L.E., Cardoso J.M., Mathias F.A., de Brito R.C. (2017). A vaccine therapy for canine visceral leishmaniasis promoted significant improvement of clinical and immune status with reduction in parasite burden. Front Immunol.

[22] Toepp A., Larson M., Wilson G., Grinnage-Pulley T., Bennett C., Leal-Lima A. (2018). Randomized, controlled, double-blinded field trial to assess Leishmania vaccine effectiveness as immunotherapy for canine leishmaniosis. Vaccine.

[23] Gradoni L., Foglia Manzillo V., Pagano A., Piantedosi D., De Luna R., Gramiccia M. (2005). Failure of a multi-subunit recombinant leishmanial vaccine (MML) to protect dogs from Leishmania infantum infection and to prevent disease progression in infected animals. Vaccine.

[24] Miret J., Nascimento E., Sampaio W., Franca J.C., Fujiwara R.T., Vale A. (2008). Evaluation of an immunochemotherapeutic protocol constituted of N-methyl meglumine antimoniate (Glucantime) and the recombinant Leish-110f + MPL-SE vaccine to treat canine visceral leishmaniasis. Vaccine.

[25] Moreno J., Nieto J., Masina S., Canavate C., Cruz I., Chicharro C. (2007). Immunization with H1, HASPB1 and MML Leishmania proteins in a vaccine trial against experimental canine leishmaniasis. Vaccine.

[26] Esch K.J., Juelsgaard R., Martinez P.A., Jones D.E., Petersen C.A. (2013). Programmed death 1-mediated T cell exhaustion during visceral leishmaniasis impairs phagocyte function. J Immunol.

[27] Gigley J.P., Bhadra R., Moretto M.M., Khan I.A. (2012). T cell exhaustion in protozoan disease. Trends Parasitol.

[28] Schaut R.G., Grinnage-Pulley T.L., Esch K.J., Toepp A.J., Duthie M.S., Howard R.F. (2016). Recovery of antigen-specific T cell responses from dogs infected with Leishmania (L.) infantum by use of vaccine associated TLR-agonist adjuvant. Vaccine.

[29] Fox C.B., Sivananthan S.J., Duthie M.S., Vergara J., Guderian J.A., Moon E. (2014). A nanoliposome delivery system to synergistically trigger TLR4 AND TLR7. J Nanobiotechnology.

[30] Raman V.S., Bhatia A., Picone A., Whittle J., Bailor H.R., O'Donnell J. (2010). Applying TLR synergy in immunotherapy: implications in cutaneous leishmaniasis. J Immunol.

